# Directional Brain Connectivity Changes Induced by Heart Rate Variability Biofeedback: A Spectral Dynamic Causal Modeling Study

**DOI:** 10.21203/rs.3.rs-10895585/v1

**Published:** 2026-09-20

**Authors:** Liangsuo Ma, Joel L. Steinberg, David Eddie, Anna Hardy, Edward A. Zuniga, Jasmin Vassileva, James M. Bjork, Andrew D Snyder, Larry D. Keen, Raul Gonzalez, Antonio Abbate, F. Gerard Moeller

**Affiliations:** Virginia Commonwealth University; Virginia Commonwealth University; Massachusetts General Hospital; Virginia Commonwealth University School of Medicine; Virginia Commonwealth University; Virginia Commonwealth University; Virginia Commonwealth University; Richmond VA Medical Center; Virginia State University; Florida International University; University of Virginia; Virginia Commonwealth University

**Keywords:** Heart Rate Variability Biofeedback, Central Autonomic Network, Dynamic Causal Modeling, resting-state fMRI, Effective Connectivity

## Abstract

Heart rate variability biofeedback (HRVB) is a non-pharmacological intervention that trains individuals to breathe at their cardiovascular resonance frequency to enhance autonomic regulation. While HRVB improves cardiovascular function across clinical conditions, the directional neural pathways through which it modulates the central autonomic network (CAN) remain poorly understood. Using a publicly available open-access dataset, we conducted a secondary analysis of effective (directional) neural connectivity (EC) data from 32 healthy participants randomized to an 8-week HRVB intervention (n = 15) or a video game Control intervention (n = 17). Pre- and post-intervention resting-state functional magnetic resonance imaging (fMRI) data were analyzed using spectral dynamic causal modeling (DCM) combined with parametric empirical Bayes to examine group differences in EC changes (ΔEC). We also evaluated whether connectivity changes correlated with changes in peripheral autonomic regulation, indexed by the standard deviation of normal-to-normal intervals (ΔSDNN). Compared with the Control group, HRVB showed strong evidence (posterior-probability = 1) of increased ΔEC from the medial prefrontal cortex to the anterior cingulate cortex and bilateral thalamus post-treatment, along with reduced ΔEC from the right hippocampus to the right ventrolateral prefrontal cortex (VLPFC). Furthermore, the regression coefficient linking hippocampus-to-VLPFC ΔEC with ΔSDNN was more (posterior-probability = 1) negative in the HRVB group than in Controls, indicating an intervention-dependent association between autonomic changes and effective connectivity. These findings indicate that HRVB alters directional fronto-limbic communication within the CAN, providing a circuit-level framework for understanding how resonance breathing modulates central autonomic control.

## INTRODUCTION

Emerging evidence supports the involvement of the central autonomic network (CAN) (Benarroch, 1993; Lamotte et al., 2021; Valenza et al., 2025) in the pathophysiology of neuropsychiatric disorders (Eddie et al., 2022; Garrett et al., 2023; Koob & Volkow, 2016). By integrating visceral, cognitive, and affective processes, the CAN detects and regulates peripheral cardiovascular activity (Eddie et al., 2022; Wareing et al., 2024). The CAN is composed primarily of the anterior cingulate cortex (ACC), medial prefrontal cortex (mPFC), orbitofrontal cortex, insula, amygdala, hippocampus, hypothalamus, striatum, thalamus, and brainstem nuclei (Valenza et al., 2025) Its role in psychiatric disorder has been attributed to its direct involvement in adaptive emotion regulation and goal-directed behavior through its bidirectional connections with cardiovascular control systems (Eddie et al., 2022; Wareing et al., 2024). Notably, a maturing literature describes how peripheral body signals (e.g. interoception) can in turn affect the brain and mood (Greenwood & Garfinkel, 2025).

This reverse, body-to-brain information flow presents a treatment opportunity, in that some bodily-derived signals can be manipulated, especially somatic signals that may be aberrant in psychiatric disorder. Heart rate variability (HRV) (Shaffer & Ginsberg, 2017) is one such signal, and is a well-established marker of the interplay between the sympathetic and parasympathetic branches of the autonomic nervous system regulated by the CAN. Within a certain range, higher HRV generally indicates more flexible and adaptive parasympathetic regulation (Heiss et al., 2021). Lower HRV is associated with impaired self-regulation and a range of psychiatric and behavioral disorders (Cattaneo et al., 2021). HRV is typically derived from the electrocardiogram or pulse photoplethysmographic recordings and analyzed with time- and frequency-domain methods (Shaffer & Ginsberg, 2017). Common time-domain measures include the root mean square of successive differences (RMSSD) and the standard deviation of normal-to-normal intervals (SDNN), while commonly used frequency-domain measures include low-frequency and high-frequency power (Shaffer & Ginsberg, 2017).

Heart rate variability biofeedback (HRVB) is a biobehavioral intervention that uses paced breathing at an individual’s cardiovascular resonance frequency to enhance autonomic regulation. This approach leverages the physiological phenomenon that the largest heart rate oscillations (smooth, sinusoidal acceleration and deceleration of heart rate across beat-to-beat intervals) occur during breathing at approximately 0.1 Hz (about six breaths/min) (Vaschillo et al., 2002). This effect is driven by respiratory sinus arrhythmia and the resonance characteristics of the cardiovascular system, mediated by the baroreflex (Vaschillo et al., 2002; Vaschillo et al., 2006). Resonance, typically between 4.5–6.5 breaths per minute, acutely increases both HRV and baroreflex activity, with optimal rate varying by individual physiology. Regular HRVB practice has been shown to enhance emotion regulation (Lehrer et al., 2020; Mather & Thayer, 2018), increase stress buffering, and reduce anxiety and depression symptoms (Goessl et al., 2017; Kenemore et al., 2024; Lin et al., 2019; Pizzoli et al., 2021). Accumulating evidence further suggests that HRVB is a promising intervention for a range of neuropsychiatric disorders (Alayan et al., 2019; Eddie et al., 2018; Eddie et al., 2014; Eddie et al., 2025; Lin et al., 2016; Penzlin et al., 2017; Teeravisutkul et al., 2019; Wieman & Eddie, 2022; Yen et al., 2022). Given that the CAN integrates autonomic, interoceptive, and emotional processes, these effects of HRVB may be mediated, at least in part, through modulation of CAN function.

Despite these benefits, the neural mechanisms underlying HRVB remain poorly understood, and only a limited number of functional magnetic resonance imaging (fMRI) studies have investigated HRVB’s effects on brain circuitry. Clarifying these neural mechanisms could illuminate why some persons respond more robustly to HRVB and could help define neurocircuit targets as secondary endpoints for mechanistic pharmacological trials designed to enhance CAN function. In particular, ascertaining *directionality* [tsl]of information flow using an analytical technique that parameterizes forward and backward connections separately can illuminate whether HRVB works by increasing top-down control signaling from the cerebral cortex or by reducing bottom-up signaling by subcortical regions. Alternatively, directional connectivity findings might inform predictive coding models that ostensibly explain how peripheral body states and signals affect the brain and cognition (Seth & Friston, 2016). In this framework, descending connections convey interoceptive predictions (top-down expectations of bodily states) while ascending connections convey interoceptive prediction errors (bottom-up visceral surprises). In this context, with a directional brain connectivity analysis, researchers can infer whether HRV biofeedback primarily lowers ascending prediction errors or sharpens the precision of top-down predictions. Finally, two cortical regions can appear strongly correlated in traditional functional connectivity analysis simply because a third, unmeasured subcortical structure—such as the Nucleus Tractus Solitarius in the brainstem or the Locus Coeruleus—is rhythmic firing and driving both regions simultaneously. Applying a model (see below) that utilizes a hidden state-space neuronal model alongside a biophysical hemodynamic model can isolate true direct neural influence between regions from shared background drivers or autonomic noise.

In a previous study with healthy adults, an 8-week HRVB program was shown to increase HRV and strengthen functional connectivity between the ventromedial prefrontal cortex (VMPFC) and limbic regions implicated in emotion regulation and autonomic control (Schumann et al., 2021). Because that study used a functional connectivity analysis, the direction of neural information flow could not be determined. Other studies (Cho et al., 2023; Nashiro et al., 2023) have similarly reported increased HRVB-related resting-state connectivity between the medial prefrontal cortex (mPFC) and left amygdala, with one study also demonstrating improved emotion-related down-regulation of somatosensory activity (Nashiro et al., 2023), although again without analysis of neural directionality. Together, these findings point to modulation of prefrontal cortical signaling as a potential pathway through which HRVB supports CAN-mediated regulation of emotion and behavior.

Building on these previous studies, the present study used spectral dynamic causal modeling (DCM) (Novelli et al., 2024) to investigate the relationship between CAN effective (directional) connectivity (EC) and HRVB using a publicly available, open-access dataset of neurotypical adults (Schumann et al., 2021). We conducted a secondary analysis of data from 32 healthy participants randomized to either an 8-week HRVB intervention (n = 15) or a Control intervention (n = 17). Spectral DCM provides more reliable estimates of connectivity than conventional functional connectivity approaches (Frassle & Stephan, 2022; Ma, Braun, Steinberg, Bjork, Martin, Keen Ii, et al., 2024) and is less susceptible to time-dependent physiological variations, such as diurnal hemodynamic fluctuations (Vaisvilaite et al., 2022). Furthermore, Bayesian empirical Bayes group-level analyses provide posterior probability estimates of effects and do not require traditional frequentist multiple-comparison corrections (Friston & Penny, 2003; Van Overwalle et al., 2019). Our primary aim was to characterize the longitudinal effects of HRVB by determining whether post- minus pre-intervention changes in EC (ΔEC) differed between intervention groups and whether associations between ΔEC and corresponding changes in autonomic function, indexed by changes in HRV, were shared across participants or specific to the HRVB intervention, as indicated by group-by-HRV interactions. Based on previous studies demonstrating increased restingstate functional connectivity between the medial prefrontal cortex (mPFC) and limbic regions of the CAN following HRVB (Cho et al., 2023; Nashiro et al., 2023; Schumann et al., 2021), we hypothesized that HRVB would strengthen top-down regulatory pathways, reflected by increased EC from the mPFC to limbic regions. Further, based on our previous findings that greater HRV was associated with weaker bottom-up EC from the amygdala to the ACC and bilateral VLPFC in healthy adults (Ma, Keen, et al., 2024), we hypothesized that HRVB would reduce these bottom-up ECs. Finally, given evidence that HRVB increases HRV, we hypothesized that changes in EC would be associated with corresponding changes in HRV, with these associations potentially reflecting either group-wide relationships or intervention-specific effects.

## METHODS AND MATERIALS

Data (Schumann et al., 2021) were obtained from OpenNeuro (Markiewicz et al., 2021) (ds003357, version 1.0.0). The current study represents an independent secondary analysis of brain effective connectivity computed from this publicly available dataset with de-identified participant information.

### Participants.

For details, see (Schumann et al., 2021). In brief, data were collected from 32 healthy participants randomly assigned to either the HRVB group (n = 15) or the control intervention group (n = 17). Available demographic characteristics are shown in Table 1.

### Intervention protocol.

For eight weeks, participants in the HRVB group completed HRVB training five times per week consisting of four sessions at home and one in the laboratory. Each session included a 5-minute resting period followed by two 11-minute training runs separated by a short break. Participants in the control intervention group followed an identical schedule but played one of three mobile video games (fast-paced platform “jump’n’run” games involving virtual running and jumping) (Schumann et al., 2021).

### HRVB methods.

For details, see (Schumann et al., 2021). In the HRVB group, participants’ current heart rate was displayed as an interpolated, smoothed curve on a smartphone screen. The goal was to maximize the amplitude of heart rate oscillations. Initially, each participant’s individual resonance frequency was estimated and used as a pacing guide for breathing during the first two weeks of training. This resonance frequency refers to the specific breathing rate (typically around 0.1 Hz or 6 breaths per minute) at which the cardiovascular system exhibits maximal heart rate oscillations due to a resonance effect between respiratory-induced changes and baroreflex-driven rhythms (Lehrer et al., 2000). Breathing at this frequency amplifies respiratory sinus arrhythmia and is believed to enhance baroreflex efficiency through repeated stimulation (Lehrer et al., 2000). In the second phase (Week 3 to Week 5), the heart rate curve began to be displayed during sessions, and participants were instructed to breathe “in phase” with the curve, i.e., inhale during ascending and exhale during descending heart rate. In the final three weeks, participants were guided to increase the amplitude of the heart rate curve to maximize HRV.

### Physiological data acquisition.

As reported in (Schumann et al., 2021), photoplethysmography (PPG) and respiratory signals were recorded during resting-state fMRI sessions using an MP150 system (BIOPAC Systems Inc., Goleta, CA, United States) at a sampling rate of 500 Hz. PPG data were acquired using an optical finger pulse sensor attached to the proximal phalanx of the participant’s left index finger. Pulse wave arrivals were identified from the first derivative of the PPG signal, which accentuates the rapid signal increase associated with pulse onset.

### HRV data processing and analysis.

HRV measures were derived from the raw PPG data, the only raw physiological recordings publicly available for download, using Kubios HRV Scientific (version 4.3.0), following a protocol adapted from Rominger et al. (Rominger et al., 2026). Pulse recordings were imported into Kubios as beat-time data. Both standard and time-varying HRV analyses were performed using the default Kubios settings. For each participant, we analyzed the first 15 minutes of the PPG recordings acquired during the resting-state fMRI scan. Frequency-domain measures were computed using Fast Fourier Transform (FFT) spectral analysis with 2-minute windows and 50% overlap. Automatic noise detection was performed using the medium threshold setting, and detected artifacts were removed. Automatic beat correction was subsequently applied, and datasets with more than 5% corrected beats were flagged for further review. To capture both overall autonomic variability and specific parasympathetic cardiac regulation, we evaluated two standard time-domain HRV metrics: the standard deviation of normal-to-normal beat intervals (SDNN) and the root mean square of successive differences (RMSSD). While both metrics reflect cardiac vagal tone during short-term resting recordings, SDNN serves as an index of total cardiac autonomic variability driven by both sympathetic and parasympathetic inputs (along with respiratory sinus arrhythmia and baroreflex activity), whereas RMSSD selectively isolates rapid, high-frequency vagally mediated parasympathetic fluctuations. To facilitate methodological consistency and replication of the heart rate variability biofeedback (HRVB) findings reported by Schumann et al. (Schumann et al., 2021), both metrics were included in the present analysis. SDNN was designated a priori as the primary HRV outcome measure and RMSSD as the secondary measure. This prioritization aligns with findings from Schumann et al. using the same dataset, wherein HRVB was associated with a significant pre- to post-intervention increase in resting SDNN, whereas resting RMSSD showed no significant change over the intervention period. Because SDNN and RMSSD typically exhibit positively skewed distributions, particularly in samples with substantial interindividual variability, both measures were natural log-transformed to reduce skewness, stabilize variance, and improve their distributional properties prior to statistical analyses. Unless otherwise specified, all SDNN and RMSSD values reported throughout the remainder of the manuscript refer to their natural log-transformed values. As the two HRV measures differed in both their means and standard deviations, they were standardized into z-scores before analyses examining the relationship between EC and HRV. Standardization improves the interpretability of regression coefficients by yielding standardized slopes, thereby facilitating comparisons of effect sizes across different linear regression models (Schielzeth, 2010).

### Magnetic resonance imaging (MRI) data acquisition.

As reported in Schumann et al. (Schumann et al., 2021), MRI scans were conducted both before the start of the intervention (pre-intervention) and after its completion (post-intervention). In brief, resting-state BOLD fMRI data were acquired with a 3 T Siemens scanner with a multiband gradient-echo echo-planar imaging (GE-EPI) sequence (TR = 484 ms, TE = 30 ms, FA = 90 deg, Multiband Factor = 8, 2.5 mm isotropic resolution, 56 slices). Approximately 15 minutes of resting-state fMRI data (up to 1,900 volumes) were collected while participants kept their eyes open; to maintain a uniform time-series length across all participants, data were truncated to 1,250 volumes per subject (corresponding to the minimum number of acquired volumes present in 3 of the 32 subjects). High-resolution anatomical T1-weighted images (MPRAGE, 1 mm isotropic resolution) were also acquired.

### Resting-state fMRI data preprocessing.

The raw structural and functional images were organized in BIDS format (Gorgolewski et al., 2016). All resting-state fMRI data passed quality control using MRIQC (Esteban et al., 2017). We subsequently preprocessed the images with fMRIPrep v20.2.1 (Esteban et al., 2019), using its default pipeline for both structural (T1-weighted) and resting-state fMRI data. Structural preprocessing included intensity non-uniformity correction using N4BiasFieldCorrection (Tustison et al., 2010), skull stripping with the ANTs-based antsBrainExtraction.sh workflow (Avants et al., 2008), and tissue segmentation into cerebrospinal fluid, white matter, and gray matter using FSL’s FAST (Zhang et al., 2001). Cortical surface reconstruction was performed with FreeSurfer’s recon-all (Dale et al., 1999), and the anatomical reference was generated using mri_robust_template (Reuter et al., 2010). Spatial normalization to MNI152NLin2009cAsym space was conducted via nonlinear registration with Advanced Normalization Tools (ANTs) antsRegistration (Avants et al., 2008), using templates accessed through TemplateFlow (Reuter et al., 2010). The brain mask was refined using a Mindboggle-based method (Klein et al., 2017) to reconcile ANTs- and FreeSurfer-derived segmentations. Functional preprocessing included motion correction using MCFLIRT (Jenkinson et al., 2002), co-registration of fMRI to T1-weighted images using boundary-based registration via FreeSurfer’s bbregister (Greve & Fischl, 2009), and normalization to MNI152NLin2009cAsym space using ANTs. Nuisance regressors were generated, including framewise displacement (Power et al., 2014), DVARS, global signals, and physiological noise components using anatomical and temporal CompCor (Behzadi et al., 2007). Additional regressors included motion derivatives, quadratic terms (Satterthwaite et al., 2013), and edge-related components (Patriat et al., 2017). Following the fMRIPrep tutorial outlined in *Andy’s Brain Book* (Jahn, 2022; doi:10.5281/zenodo.5879293), we applied two additional post-processing steps: spatial smoothing using a 4 mm FWHM kernel and scaling of the BOLD signal to a mean of 100. Following (Ma, Braun, Steinberg, Bjork, Martin, Keen, et al., 2024), we further applied high-pass temporal filtering with a cutoff frequency of 0.01 Hz.

### DCM analysis.

Spectral DCM (Friston et al., 2014), as implemented in SPM25, was used to analyze the HRVB resting-state fMRI dataset. For consistency, the 12-node model architecture and node specification used in the present study directly replicate the DCM framework previously used by Ma et al. (Ma, Keen, et al., 2024) for testing the linear relationship between resting-state EC and HRV measures, wherein each node was defined as an 8-mm radius sphere. These nodes were the left VLPFC, right VLPFC, mPFC, left middle temporal gyrus (MTG), right MTG, supracallosal ACC, right anterior insula, right amygdala, right hippocampus, left thalamus, right thalamus, and right supramarginal gyrus (SMG). The Montreal Neurological Institute (MNI) coordinates of the center of each node are provided in Table 2. For each participant and each MRI session (Pre- and Post-intervention), an initial putative fully connected DCM was specified, allowing each node to influence itself and all other nodes. For each participant, DCM model estimation pruned connections that showed zero EC strength. Group-level EC analyses were conducted using the parametric empirical Bayes (PEB) framework (Friston et al., 2016). These analyses evaluated four primary effects: (i.a) whether pre-intervention EC parameters showed strong evidence of being non-zero across all participants; (i.b) whether these baseline EC parameters were reliably associated with pre-intervention heart rate variability (HRV) measures (potential replication of individual difference findings in (Ma, Keen, et al., 2024); (ii.a) whether post-minus pre-intervention EC changes (ΔEC) showed strong evidence of group differences (HRVB vs. Control); and (ii.b) whether the relationship between ΔEC and post- minus pre-intervention changes in HRV measures (ΔHRV) demonstrated a reliable (and non-specific) main effect of time on HRV across both groups combined or a reliable group-by-time interaction effect on HRV.

Analyses (i.a) and (i.b) were conducted following the procedures described by Ma et al. (Ma, Keen, et al., 2024). For analyses (ii.a) and (ii.b), ΔEC was quantified using a within-subject PEB approach for each of the 32 participants. First, the first-level DCMs for the pre- and post-intervention sessions were estimated separately for each subject. A within-subject PEB model was then specified to evaluate the linear contrast between sessions (post-intervention minus pre-intervention). This procedure systematically reduced the session-specific DCMs to a single, optimized PEB model per participant, yielding a vector of estimated ΔEC parameters for each individual. These participant-specific EC changes were subsequently carried forward to the group-level PEB analyses for (ii.a) and (ii.b). To address the group-level objectives, the participant-specific EC change (ΔEC) parameters obtained from the first-level within-subject PEBs were carried forward into two distinct second-level PEB models. For Analysis (ii.a), which evaluated group differences in EC changes, a two-regressor design matrix was configured consisting of a grand mean intercept (Regressor 1) and a mean-centered group main effect coded as 1 for HRVB and – 1 for Control (Regressor 2) groups. For Analysis (ii.b), which evaluated the linear relationships between EC changes and HRV measures, a four-regressor design matrix was implemented. This matrix included the grand mean intercept (Regressor 1), the group main effect (Regressor 2), a continuous vector of mean-centered ΔHRV scores evaluating the overall relationship between ΔEC and ΔHRV across all participants (Regressor 3), and an element-wise product interaction term (Regressor 2 × Regressor 3) explicitly testing whether the relationship between ΔEC and ΔHRV was modulated by group membership. For all these analyses, Bayesian model averaging (BMA) (Penny et al., 2010) was subsequently performed to identify robust and reliable group level results. The reliability of findings was evaluated using Bayesian posterior probability (Bayesian-PP). A Bayesian-PP above 0.95 was interpreted as strong evidence (equivalent to a Bayes factor of 20). There are two kinds of ECs in DCM: between-node EC and self-connection. According to Zeidman et al. (Zeidman et al., 2019), a greater self-connection parameter value indicates stronger self-inhibition, rendering the region less responsive to inputs from the network. Conversely, a lower value reflects reduced self-inhibition, thereby increasing the region’s sensitivity to network inputs. These unitless self-connection parameters can be converted into values with units of hertz (constrained to be negative). However, in this study, we retained the unitless self-connection parameter values to be consistent with the interpretive framework described above.

## RESULTS

### HRV Results.

Table 3 summarizes resting HRV measures at pre-intervention, post-intervention, and the corresponding changes (ΔHRV) from pre- to post-intervention in the HRVB and Control groups. In the HRVB group, resting SDNN increased from pre- to post-intervention (i.e., ΔSDNN > 0, Student’s t-test, uncorrected p = 0.02; Bonferroni-corrected p = 0.04 across the two primary HRV outcome measures). As shown in Table 3, all remaining comparisons yielded non-significant results (p > 0.05). Statistical analyses using the log-transformed HRV measures yielded similar results (Table 3).

### Baseline EC and its Relationship with HRV (Analyses i.a and i.b).

**Figure 1:**
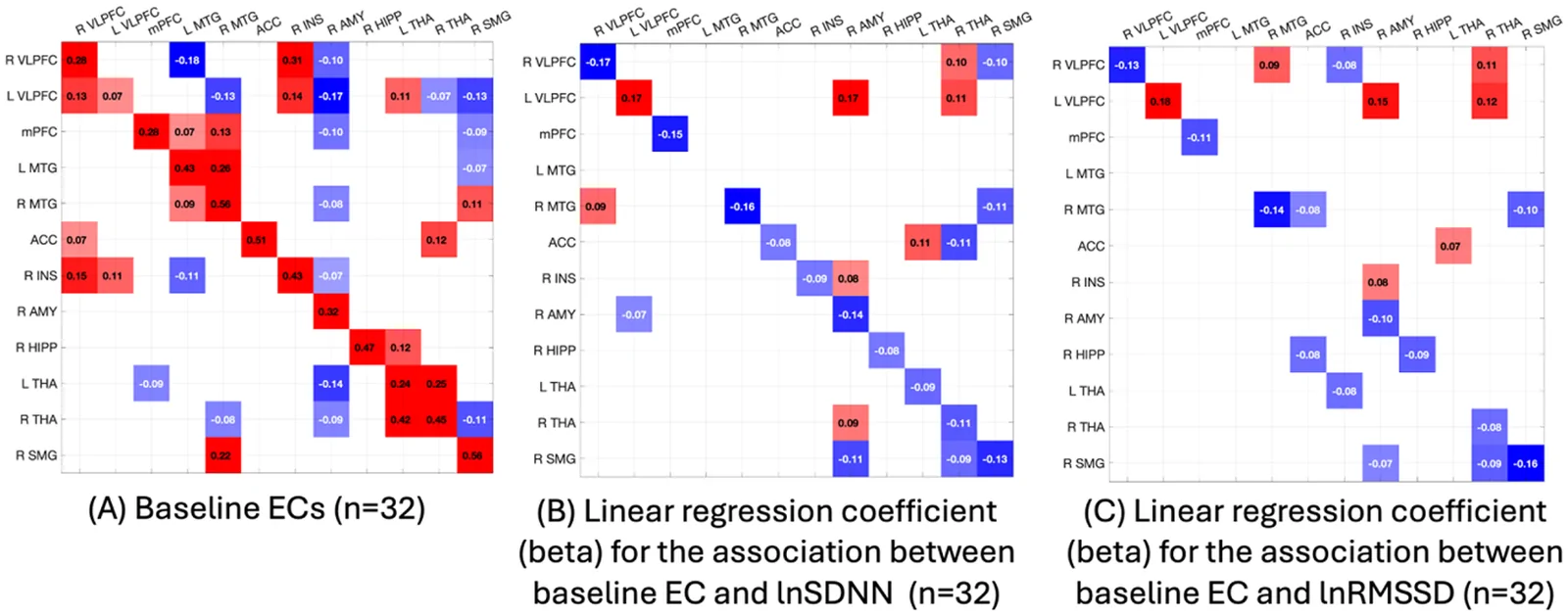
Baseline (pre-intervention) effective connectivity (EC) (Panel A); linear regression coefficients (beta) for the association between baseline EC and SDNN (Panel B); and linear regression coefficients (beta) for the association between baseline EC and RMSSD (Panel C). In Panel A, off-diagonal ECs are expressed in hertz (Hz), whereas on-diagonal (self-)connections are unitless. Each matrix element represents the strength and direction of the EC from the source region (column) to the target region (row). Panel B and Panel C display the corresponding regression coefficients (beta; unitless) from the PEB model relating baseline EC to SDNN and RMSSD respectively. In both panels, ECs or regression coefficients with a Bayesian posterior probability (Bayesian-PP) ≤ 0.95 are set to zero (white cells). Non-zero ECs or regression coefficients (beta) with Bayesian-PP > 0.95 are displayed using both numerical values and a color map, with warm colors indicating positive values and cool colors indicating negative values. Abbreviations: ACC = anterior cingulate cortex; AMY = amygdala; HIP = hippocampus; INS = insula; mPFC = medial prefrontal cortex; MTG = middle temporal gyrus; SMG = supramarginal gyrus; THA = thalamus; VLPFC = ventrolateral prefrontal cortex.

At baseline, the group-level PEB analysis revealed that 39 ECs showed strong evidence of non-zero across all participants (Bayesian-PP > 0.95; Analysis i.a), as illustrated in Fig. 1A. Furthermore, 24 baseline ECs exhibited a reliable linear relationship with the SDNN measure (Bayesian-PP > 0.95; Analysis i.b). The linear regression coefficients (betas) characterizing these 24 relationships are presented in Fig. 1B. Similar linear regression analysis evaluated the relationships between baseline EC and RMSSD; these results are provided in Fig. 1C.

### Effects of HRVB on ECs and their linear relationship with HRV.

**Figure 2:**
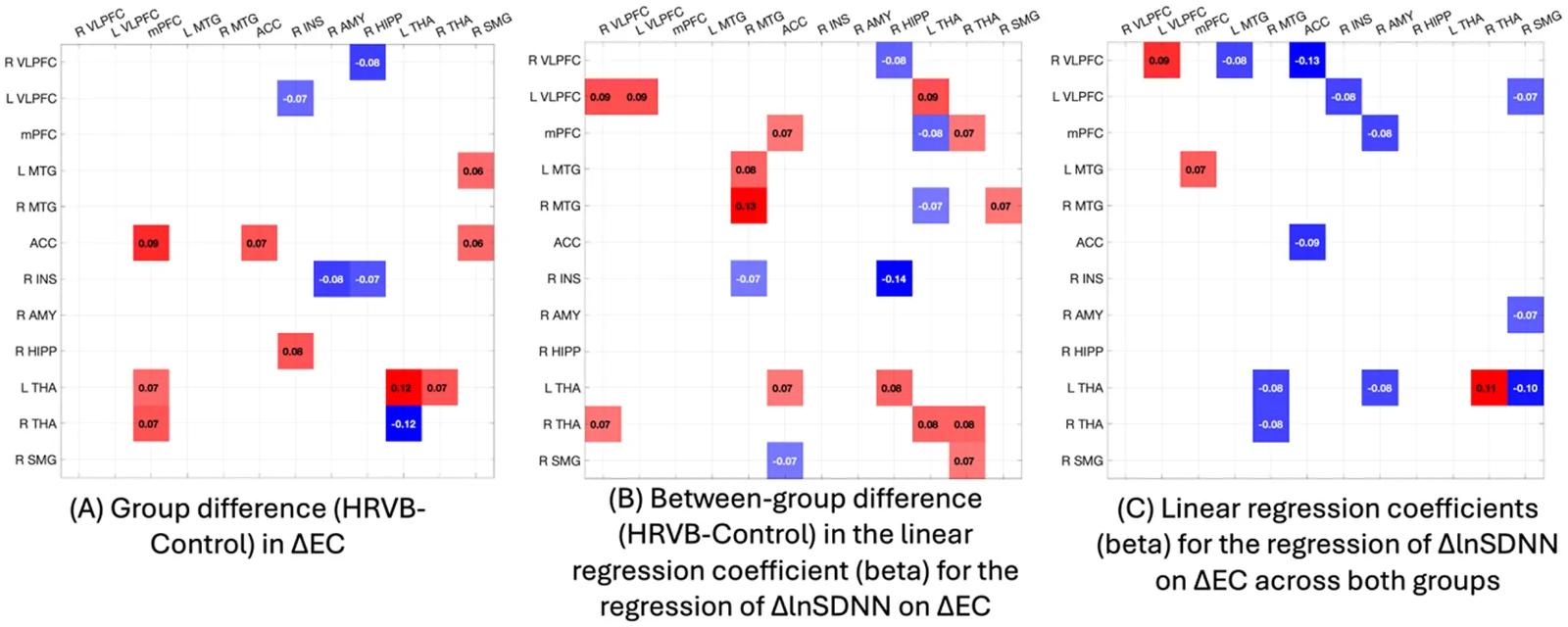
Group difference (HRVB-Control) in ΔEC (Panel A); between-group difference (HRVB-Control) of the linear regression coefficients (betas) for the regression of ΔSDNN on ΔEC (Panel B); and linear regression coefficients (betas) for the regression of ΔSDNN on ΔEC across both groups (Panel C). In Panel A, off-diagonal ΔECs are expressed in hertz (Hz), whereas on-diagonal (self-) connections are unitless. Each matrix element represents the strength and direction of the ΔEC from the source region (column) to the target region (row). Panel B and Panel C display the corresponding regression coefficients (beta; unitless) from the PEB model relating ΔEC to ΔSDNN for both groups combined (Panel B) and group difference (HRVB-Control). In all panels, ΔECs or regression coefficients with a Bayesian posterior probability (Bayesian-PP) ≤ 0.95 are set to zero (white cells). Non-zero ΔECs or regression coefficients (beta) with Bayesian-PP > 0.95 are displayed using both numerical values and a color map, with warm colors indicating positive values and cool colors indicating negative values. Abbreviations: ACC = anterior cingulate cortex; AMY = amygdala; HIP = hippocampus; INS = insula; mPFC = medial prefrontal cortex; MTG = middle temporal gyrus; SMG = supramarginal gyrus; THA = thalamus; VLPFC = ventrolateral prefrontal cortex.

**Figure 3:**
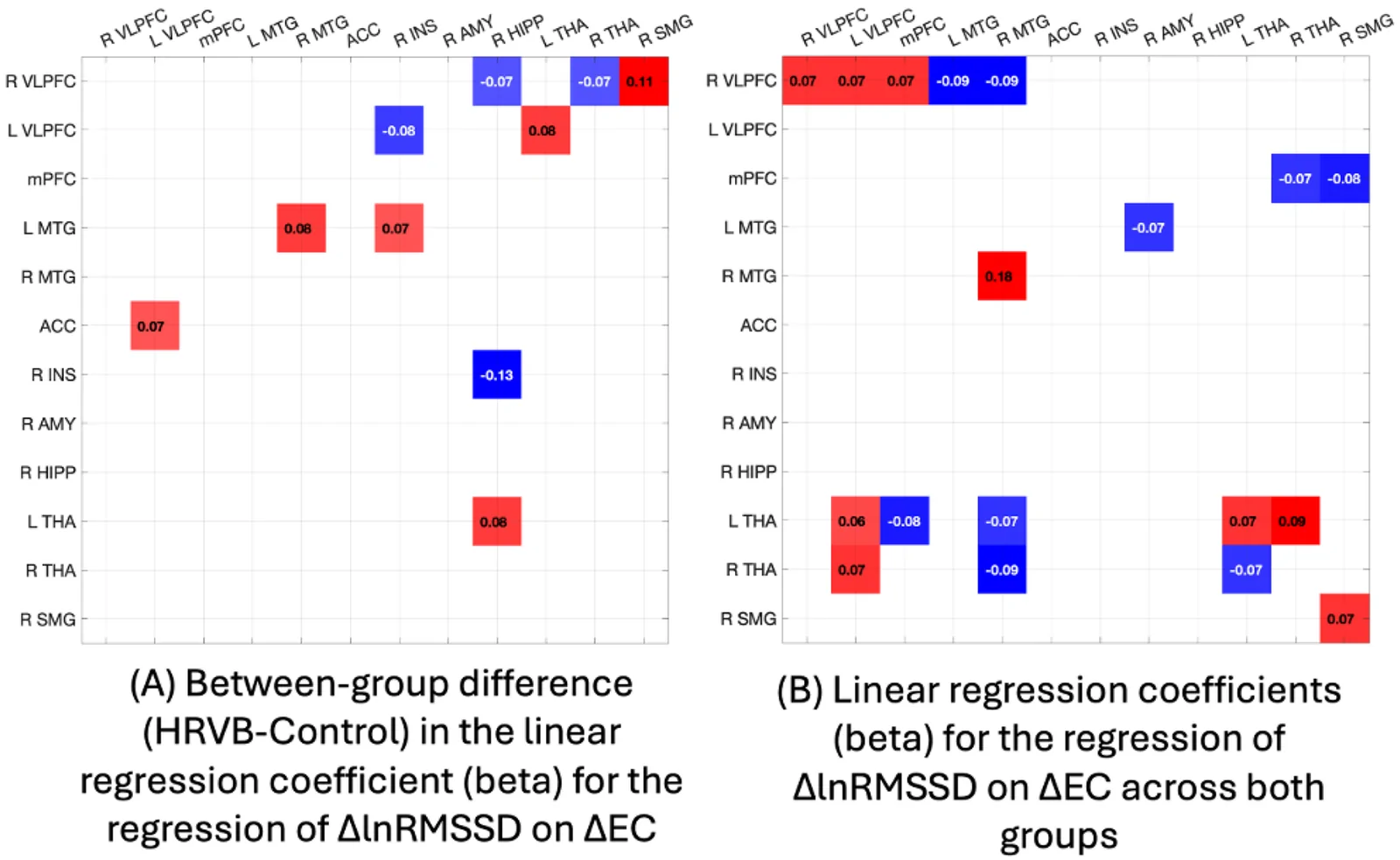
Between-group difference (HRVB-Control) of the linear regression coefficients (betas, unitless) for the regression of ΔRMSSD on ΔEC (Panel A); and linear regression coefficients (betas) for the regression of ΔRMSSD on ΔEC across both groups (Panel B). Each matrix element represents the strength of the beta for each EC with the direction from the source region (column) to the target region (row). In both panels, regression coefficients with a Bayesian posterior probability (Bayesian-PP) ≤ 0.95 are set to zero (white cells). Non-zero regression coefficients (beta) with Bayesian-PP > 0.95 are displayed using both numerical values and a color map, with warm colors indicating positive values and cool colors indicating negative values. Abbreviations: ACC = anterior cingulate cortex; AMY = amygdala; HIP = hippocampus; INS = insula; mPFC = medial prefrontal cortex; MTG = middle temporal gyrus; SMG = supramarginal gyrus; THA = thalamus; VLPFC = ventrolateral prefrontal cortex.

The group-level PEB analysis for group differences (Analysis ii.a) revealed that from pre- to post-intervention, the HRVB group exhibited reliable changes in 15 EC parameters (including the ECs from mPFC to ACC and bilateral thalamus and the EC from right hippocampus to right VLPFC) compared to the Control group (Bayesian-PP > 0.95; Fig. 2A). Analysis ii.b identified 20 ΔEC parameters (including the ECs from right hippocampus to right VLPFC and right anterior insula) that exhibited a reliable group-by-ΔSDNN interaction effect (Bayesian-PP > 0.95). The differences of the linear regression coefficients (betas) between groups, characterizing these interaction effects, are displayed in Fig. 2B. Furthermore, for the 124 ECs that did not show a reliable group-by-ΔSDNN interaction effect in Analysis ii.b, 14 ΔECs across all participants demonstrated a reliable linear relationship with ΔSDNN (Bayesian-PP > 0.95), with the corresponding linear regression coefficients (betas) presented in Fig. 2C. Parallel ii.b analyses were also conducted using ΔRMSSD, with the corresponding results presented in Fig. 3. Similar to ΔSDNN, ECs from right hippocampus to right VLPFC and right anterior insula exhibited a reliable group-by-ΔRMSSD interaction effect (Bayesian-PP > 0.95).

## DISCUSSION

The present study used spectral DCM to provide the first directionally specific characterization of HRVB modulation of ECs within the CAN. Our principal findings demonstrate strong evidence (Bayesian-PP > 0.95) that an 8-week HRVB intervention affects the strength of fronto-limbic directional neural connectivity. Specifically, compared to the Control intervention, HRVB reliably enhanced top-down influence from the mPFC to the ACC and to the bilateral thalamus. Concurrently, HRVB attenuated bottom-up signaling from limbic regions, resulting in lower ECs from the right hippocampus to the right VLPFC and to the right anterior insula. These HRVB-related changes in subcortical-to-cortical signaling pathways correlated with changes in ΔSDNN. Below, we discuss the potential mechanisms and implications of the HRVB-related EC changes that appear most mechanistically relevant.

### ECs from mPFC to ACC and bilateral thalamus.

Compared with the Control intervention, the HRVB intervention increased ECs from the mPFC to the ACC and bilateral thalamus (Fig. 2A), supporting Hypothesis 1. Specifically, ECs from the mPFC to the ACC (EC = 0.1701 Hz, Bayesian PP = 1), left thalamus (EC = 0.1173 Hz, Bayesian PP = 1), and right thalamus (EC = 0.1558 Hz, Bayesian PP = 1) were positive in the HRVB group, whereas these ECs were not reliably different from zero in the Control group (EC = 0 Hz, Bayesian PP = 0 for all three connections). This pattern suggests that HRVB was associated with the emergence of positive top-down influence from the mPFC to cingulate and thalamic regions, rather than simply strengthening an already established directional influence. The role of the mPFC identified in the present study is consistent with previous findings implicating this region in the neural effects of HRVB (Cho et al., 2023; Nashiro et al., 2023; Schumann et al., 2021). However, contrary to Hypothesis 3, longitudinal changes in these top-down pathways were not associated with changes in HRV, as evidenced by the absence of an intervention-specific group-by-HRV interaction (Fig. 2B) and a group-wide association across participants (Fig. 2C). This dissociation suggests that enhancement of fronto-cingulate and fronto-thalamic ECs may represent a central mechanism through which HRVB alters brain function that is not directly coupled to changes in peripheral autonomic output.

### EC from right hippocampus to right VLPFC.

Although this pathway was not included in our a priori hypotheses, the DCM analysis revealed that ΔEC in this pathway was lower in the HRVB group than in the Control group (Fig. 2A). Specifically, the right hippocampus-to-right VLPFC EC was negative in the HRVB group (EC = − 0.0939 Hz, Bayesian PP = 1), whereas it was not reliably different from zero in the Control group (EC = 0 Hz, Bayesian PP = 0). This pattern suggests that HRVB shifted hippocampus-to-VLPFC signaling toward reduced or negative effective influence, rather than simply weakening an already established positive connection. The regression coefficient relating ΔEC to ΔSDNN was more negative in the HRVB group than in the Control group (Fig. 2B). Similar results were also found in the analyses related to RMSSD (Fig. 3). Post-hoc simple linear regression analyses further showed that right hippocampus-to-right VLPFC EC was negatively associated with both SDNN (beta = − 0.0935, Bayesian PP = 1) and RMSSD (beta = − 0.0780, Bayesian PP = 1) in the HRVB group, whereas no reliable associations were observed in the Control group (beta = 0, Bayesian PP = 0 for both SDNN and RMSSD). These findings clarify that the Group × HRV interaction reflected the emergence of a negative EC–HRV relationship following HRVB, rather than simply a difference in the magnitude of an association that was present in both groups. These results indicate an intervention-specific strengthening of the relationship between HRV and this fronto-limbic pathway. The hippocampus is involved in contextual memory and stress-related processing (63), whereas the VLPFC plays roles in cognitive control (64) and emotion regulation (65). Thus, the observed reduction in hippocampus-to-VLPFC EC following HRVB may reflect altered communication between memory-related and prefrontal regulatory systems. Together, these findings suggest that HRVB is associated with changes in both peripheral autonomic regulation and fronto-limbic EC, providing insight into possible neural mechanisms through which HRVB may influence the CAN.

### EC from right hippocampus to right anterior insula.

The DCM analysis revealed that ΔEC in this pathway was lower in the HRVB group than in the Control group (Fig. 2A). The regression coefficient relating ΔEC to ΔSDNN (Fig. 2B) was lower in the HRVB group than in the Control group. Similar results were also observed in the analyses involving RMSSD (Fig. 3). Post-hoc simple linear regression analyses indicated that right hippocampus-to-right anterior insula EC was negatively associated with both SDNN (beta = − 0.1431, Bayesian PP = 1) and RMSSD (beta = − 0.1180, Bayesian PP = 1) in the Control group, whereas no reliable associations were observed in the HRVB group (β = 0, Bayesian PP = 0 for both SDNN and RMSSD). Thus, the Group × HRV interaction reflected a negative EC–HRV relationship that was present in the Control group but absent following HRVB. Although this EC was not hypothesized a priori, these findings indicate that the relationship between HRV and hippocampus-to-anterior insula EC differed between the HRVB and Control groups. The hippocampus and anterior insula are components of neural circuits supporting contextual memory processing (Anand & Dhikav, 2012) and interoceptive awareness (Wang et al., 2019), respectively. The loss of this EC–HRV association following HRVB may suggest that the intervention altered the coupling between peripheral autonomic regulation and hippocampal–interoceptive signaling. These findings suggest that HRVB may modify the coupling between contextual memory-related and interoceptive processing within the CAN.

### ECs from right amygdala to ACC and bilateral VLPFC.

Contrary to Hypothesis 2, changes in these ECs did not differ significantly between the HRVB and Control groups (Fig. 2A). For Hypothesis 3, analyses examining the associations between changes in these three ECs and changes in the two log-transformed HRV measures revealed no significant Group × HRV interactions or group main effects. Thus, there was no evidence that HRVB-related changes in these amygdala-to-prefrontal and amygdala-to-cingulate pathways were associated with changes in HRV.

### Differential HRV Responses to HRVB.

A key finding of our analysis, consistent with Schumann et al. (Schumann et al., 2021), was the differential response of the primary (SDNN) and secondary (RMSSD) HRV measures to the HRVB intervention. Specifically, resting SDNN increased from pre- to post-intervention in the HRVB group, whereas RMSSD did not show a significant change. Although both SDNN and RMSSD are widely utilized time-domain indices of HRV, they reflect distinct physiological mechanisms during short-term recordings. RMSSD is driven almost exclusively by vagally mediated, beat-to-beat parasympathetic outflow via the nucleus ambiguous. In contrast, short-term SDNN reflects total autonomic variance, capturing a broader integration of parasympathetic tone, sympathetic influences, and critically, baroreflex sensitivity and resonance phenomena induced by slow breathing. Because HRVB directly trains baroreflex function and respiratory-cardiac coupling at the individual’s resonance frequency (~ 0.1 Hz), its primary physiological effect is an enhancement of low-frequency oscillations and overall baroreflex-mediated autonomic amplitude. Consequently, the observed post-intervention increase in resting SDNN without a corresponding change in resting RMSSD suggests that HRVB may primarily adapt baroreflex gain and autonomic flexibility rather than altering tonic, high-frequency basal parasympathetic tone.

### Consistency in results across studies.

The HRV measures in the present study were calculated using Kubios HRV Scientific, whereas Schumann et al. (2021) computed HRV measures using in-house MATLAB code. Although the absolute HRV values (e.g., means and standard deviations) differed somewhat between the two studies, the overall findings were highly consistent, with both analyses demonstrating an increase in SDNN from pre- to post-intervention following HRV biofeedback. Similarly, of the 39 non-zero baseline ECs identified in the present study (Fig. 1A), 29 (74%) overlapped with those reported by Ma et al. (2024), which used the same DCM network to investigate the relationship between EC and HRV in healthy persons. This substantial overlap indicates good reproducibility of baseline CAN effective connectivity across independent studies of healthy subjects. However, the previously reported negative associations between RMSSD and ECs from the right amygdala to the ACC and bilateral VLPFC (Ma, Keen, et al., 2024) were not replicated at baseline, possibly reflecting differences in participant characteristics between the two studies.

### Limitations.

First, the DCM analysis was restricted to a limited number of regions within the central autonomic network, as discussed in our previous study (Ma, Keen, et al., 2024). Second, the present study included a relatively small sample size. Nevertheless, the baseline EC showed substantial consistency with our previous study (Ma, Keen, et al., 2024) with a completely different group of subjects, with approximately 74% of non-zero ECs overlapping between the two studies. Furthermore, recent empirical work has demonstrated that resting-state DCM can provide reliable estimates even with relatively small sample sizes (e.g., *n* = 20), particularly for the stronger ECs (Ma, Braun, Steinberg, Bjork, Martin, Keen, et al., 2024).

### Summary.

This study contributes to the understanding of brain–heart interactions by demonstrating that HRVB may affect directional neural communication within the CAN. Using spectral DCM, we demonstrated that an 8-week HRVB intervention shifts fronto-limbic connectivity toward greater top-down cortical influence, evidenced by increased change of ECs from the mPFC to the ACC and bilateral thalamus. Concurrently, HRVB modulates subcortical circuits by reducing bottom-up EC from the right hippocampus to the right VLPFC. These EC changes were strongly coupled with improvements in HRV, as changes in this hippocampus-to-VLPFC pathway were negatively associated with ΔSDNN in an intervention-dependent manner. The findings of this study advanced our understanding of possible brain neural effects of HRVB intervention and suggest possible neurocircuit targets for future research into autonomic dysregulation. However, we caution that these results may not be replicated in clinical samples, where individuals may be undergoing HRVB to remedy poor emotional regulation.

## Figures and Tables

**Table 1:** Demographic information (i.e., age, sex, and education) is presented for the HRVB and Control groups. Group differences in sex and age were tested using chi-square test and Student t-test respectively. All p-values are two-tailed.

Parameter	HRVB (n = 15)	Control (n = 17)	Statistical results of group difference
Age [years] mean and standard deviation (range)	30.2 ± 7.9 (22 to 52)	28.2 ± 9.8 (18 to 53)	*t* = 0.53, df = 30, *p* = 0.63
Sex	8 F, 7 M	9 F, 8 M	*χ^2^*=0, *df* = 1, *p* = 0.98

**Table 2:** The MNI coordinates of all DCM nodes.

DCM nodes	MNI coordinates (x, y, z) mm
Left ventrolateral prefrontal cortex	−5420-2
Right ventrolateral prefrontal cortex	5420-2
Medial prefrontal cortex	052 – 12
Left middle temporal gyrus	−52-6822
Right middle temporal gyrus	54-6022
Anterior cingulate cortex	03624
Right anterior insula	4620-10
Right amygdala	220 – 16
Right hippocampus	24-20-14
Left thalamus	−4-188
Right thalamus	6-168
Right SupraMarginal gyrus	60-4626

**Table 3:** Heart rate variability (HRV) and heart rate (HR) measures at pre-intervention (Pre), post-intervention (Post), and the corresponding changes (Δ) = Post minus Pre in the HRV biofeedback (HRVB) and Control groups. Note: RMSSD = root mean square of successive differences between normal-to-normal heart beat intervals; SDNN = standard deviation of normal-to-normal heart beat intervals; lnSDNN = natural log-transformed SDNN; and lnRMSSD = natural log-transformed RMSSD.

	HRVB group (n = 15)				Control group (n = 17)				Between- group difference in Δ
	Pre	Post	Δ	Sig.	Pre	Post	Δ	Sig.	
Raw SDNN (ms)	44.62 ± 18.66	51.99 ± 18.90	7.38 ± 18.90	t = 3.03, df = 14, p = 0.009	52.18 ± 28.97	48.01 ± 27.97	−4.17 ± 18.44	t = 0.93, df = 16, p = 0.37	t = 2.18, df = 30, p = 0.04
Raw RMSSD (ms)	47.36 ± 26.10	51.50 ± 19.11	4.14 ± 17.01	t = 0.92, df = 14, p = 0.37	57.28 ± 33.73	47.84 ± 25.06	−9.44 ± 26.00	t = 1.50, df = 16, p = 0.15	t = 1.72, df = 30, p = 0.095
lnSDNN	3.71 ± 0.42	3.89 ± 0.38	0.17 ± 0.25	t = 2.63, df = 14, p = 0.02	3.81 ± 0.58	3.74 ± 0.51	−0.06 ± 0.38	t = 0.65, df = 16, p = 0.52	t = 1.99, df = 30, p = 0.06
lnRMSSD	3.72 ± 0.56	3.88 ± 0.38	0.16 ± 0.35	t = 1.77, df = 14, p = 0.10	3.90 ± 0.57	3.74 ± 0.52	−0.15 ± 0.46	t = 1.34, df = 16, p = 0.20	t = 2.12 df = 30, p = 0.04

## Data Availability

A MATLAB implementation of the dynamic causal modeling (DCM) approach is available as open-source code in the Statistical Parametric Mapping software package (https://www.fil.ion.ucl.ac.uk/spm/). The data used in this study can be downloaded from the OpenNeuro (https://openneuro.org/).
